# Asthma diagnosis and treatment – 1011. OZAC- a herbal medicine for bronchial asthma

**DOI:** 10.1186/1939-4551-6-S1-P11

**Published:** 2013-04-23

**Authors:** Hari Kishan Gonuguntla, Parankusam Rao, Bhukya Naik

**Affiliations:** 1 Pulmonology, Katuri Medical College & Hospital, Guntur, India

## Background

To assess the efficacy of a herbal medicine made up of ocimum sanctum,zingiber officinale,azadirachta indica,curcuma longa in a titrated combination tablet prepared by 'IMMIS’ labs, India for bronchial asthma patients.

## Methods

100 subjects suffering from bronchial asthma were enrolled for this study from 2012 January to February, after obtaining consent of the participants and ethical committee approval. The bronchial asthma severity was graded as severe pefr <200ltrs\Mt, moderate 200-300ltrs\Mt, mild 300-400ltrs\Mt. For this study moderate and mild categories were selected. Furthermore 20% (10% each of moderate and mild) were considered as control.

All the ingredients of OZAC together have the pharmacological action of bronchodilatation, antiallergic ,mucolytic and antihistaminic properties. The entry samples were assessed with Wrighst peak flow meter before and after the observation period of three weeks, besides the subjective evaluation. As and when required they were administered long acting fluticasone+fometerol (FF), short acting salbutamol (SI) aerosol with an internal spacer TOPS (trans oro pharyngeal spacer) and doxophylline tabs (400mg).

## Results

1. The entire sample consisted of 70 males and 30 females within the age range of15 to 80 years.

2. The mild group (45%) required 2tabs of ozac twice daily and FF inhaler one puff daily. The improvement in the pefr (15%)-pre and post treatment was significant (p<.001).

3. The moderate group had 2tabs of ozac twice daily+FF BID+ Salbutamol sos. The reversibility was significant (p<.001).

4. The control group moderate and mild required FF+SI+doxophylline (400mg bid).

## Conclusions

Ozac has distinct advantage in the treatment of bronchial asthma. The unique properties of its ingredients makes it an effective adjuvant to the asthma medication.

1. It reduces the need of the inhalers and other bronchodilators

2. It induces confidence in the users

3. It is cost effective and can be an effective adjuvant

4. There are no reported side effects.

